# Supplementary material for: Homologs of Ancestral CNNM Proteins Affect Magnesium Homeostasis and Circadian Rhythmicity in a Model Eukaryotic Cell
Source: Int J Mol Sci. 2023 Jan 23;24(3):2273. doi: 10.3390/ijms24032273 (PMC9916543; doi:10.3390/ijms24032273)
Supplement: Supplementary file 1 [file ijms-24-02273-s001.zip › ijms-2144695-supplementary.pdf]

Supplementary Materials

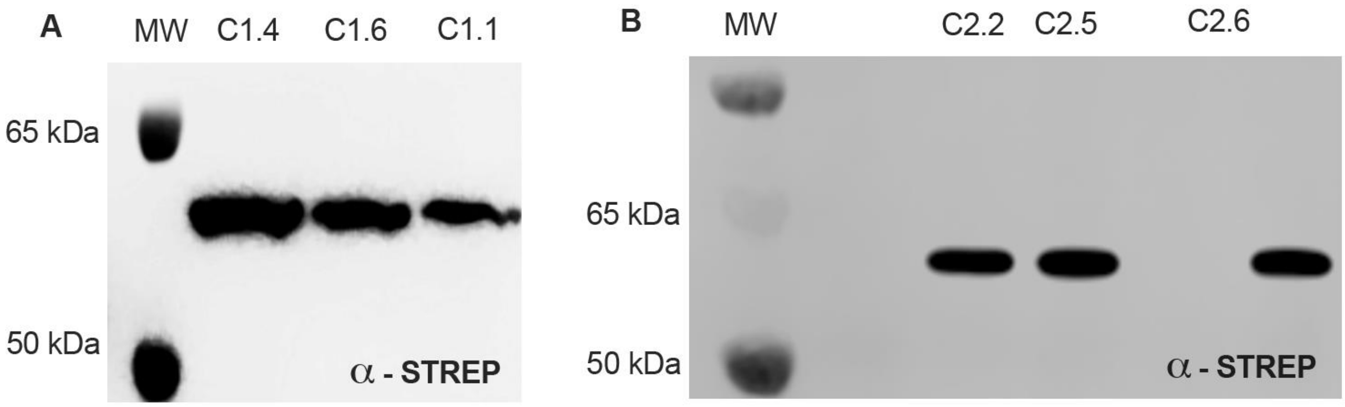

Figure S1. Immunoblotting analyses against STREP tag to validate protein levels of the CNNM1ox (**A**) and CNNM2ox (**B**) lines.

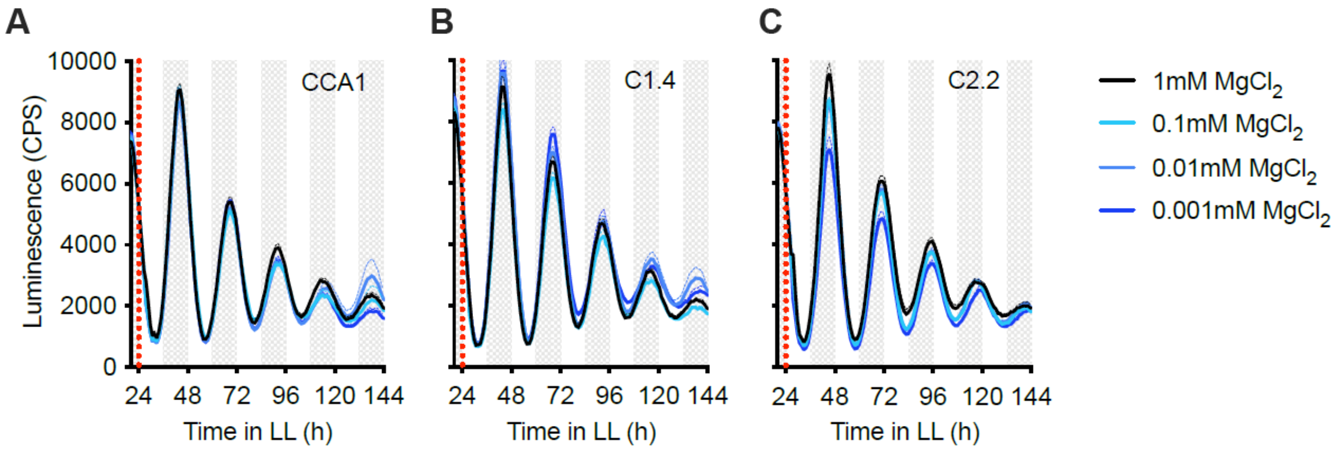

Figure S2. Luminescent traces of CCA1-LUC under LL for the parent line (**A**), CNNM1ox (**B**) and CNNM2ox. (**C**) lines subjected to low-Mg medias. Grey shade areas represent subjective night periods. CPS = Counts per second.

Table S1. Accession numbers and protein information of CNNM proteins found in eukaryotes.

Species	Taxa ID	Protein number/name	ncbi accession number	Other
Thalassiosira pseudonana	296543	**1**	XP_002291595.1	
		**2**	XP_002292385.1	
		**3**	XP_002292530.1	
Chondrus crispus	2769	**1**	XP_005712858.1	
		**2**	XP_005716511.1	
		**3**	XP_005718375.1	
Emiliania huxleyi	280463	**1**	XP_005767214.1	
		**2**	XP_005770560.1	
		**3**	XP_005773754.1	
Guillardia theta	70448	CNNM1	XP_003079248.2	ostta05g01490
		CNNM2	XP_003081913.1	ostta11g01030
Homo sapiens	9606	CNNM1	NP_065081.2	
		CNNM2	NP_060119.3	
		CNNM3	NP_060093.3	
		CNNM4	XP_047299867.1	
Drosophila melanogaster	7227	**1**	NP_001104390.2	
Crassostrea gigas	29159	1	XP_011444497.2	
		2	XP_011456345.2	
		3	XP_011456353.2	
Salpingoeca rosetta	946362	**1**	XP_004994776.1	
		**2**	XP_004994099.1	
Amphimedon queenslandica	400682	**1**	XP_019851308.1	
		**2**	XP_019848829.1	
		**3**	XP_003384720.1	
Arabidopsis thali-ana	3702	MGR1	NP_193160.3	AT4G14240
		MGR2	NP_193159.3	AT4G14230
		MGR3	NP_171826.2	AT1G03270
		MGR4	NP_175166.2	AT1G47330
		MGR5	NP_200091.2	AT5G52790
		MGR6	NP_195096.2	AT4G33700
		MGR7	NP_001324217.1	AT2G14520
		MGR8	NP_187914.1	AT3G13070
		MGR9	NP_175989.2	AT1G55930
Medicago truncatula	3880	CBS1	AJI77175.1	
Neurospora Crassa	367110	1	XP_960440.3	
Botrytis cinerea	332648	***1***	XP_001556285.1	
Saccharomyces cerevisiae	559292	MAM3	NP_014581.1	
Methanoculleus thermophilus	2200	CorC	WP_066956488.1	
Gloeomargarita lithophora	1188229	CorA	APB35145.1	

Table S2. Primers used for CNNM1 and CNNM2 gene cloning. FW = Forward, RV = Reverse.

	DNA sequence (5′ ŕ3′)
FW CNNM1	CATCCTAGGATGGCCTCGACGCTCGATGCGT
RV CNNM1	CATCCTAGGATCCTCCGTGTCTGCACTTGGC
FW CNNM2	CATCCTAGGATGGCGGCGGTGACGTTCGCGAGCC
RV CNNM2	CATCCTAGGCAACGCGCGAGGAACGATCGGTTTT
